# Higher Residence Attachment and Religiosity Are Associated With Less Depressive Symptoms After Terror Event Exposure

**DOI:** 10.3389/fpsyg.2021.760415

**Published:** 2021-12-09

**Authors:** Liat Korn, Miriam Billig, Gil Zukerman

**Affiliations:** ^1^Department of Health Management Systems, Ariel University, Ariel, Israel; ^2^Department of Sociology and Anthropology, Eastern R&D Center, Ariel University, Ariel, Israel; ^3^Department of Communication Disorders, Ariel University, Ariel, Israel

**Keywords:** terror event exposure, residence attachment, depression symptoms, religion, psychosomatic symptoms, undergraduate students

## Abstract

**Introduction:** We examined how community type, residence attachment, and religiosity contribute to resilience to depressive symptoms, psychosomatic complaints, residential stress, and avoidance behavior among students exposed to terror.

**Methods:** Undergraduate students from Ariel University (*N* = 1,413; 62.7% females; *M*_age_ = 26.5; SD = 6.03) completed a self-report questionnaire on socio-demographics, terror exposure, place attachment, and depressive/psychosomatic symptoms. Participants were divided into three residential groups: “Ariel,” “Small settlement communities in Judea and Samaria” or “Other places in Israel.”

**Results:** Participants from small settlement communities in Judea and Samaria showed significantly fewer depressive symptoms and greater adjustment– less avoidance, psychosomatic symptoms, and residential stress– compared to those living in Ariel or other places in Israel, despite significantly higher exposure to terror.

**Conclusion:** Greater religiosity and residence attachment may protect against depressive symptom development following terror exposure. Secular, temporary residents living in highly terror-exposed areas should be targeted for community strengthening interventions.

## Introduction

Exposure to man-made traumatic events, particularly acts of terror (Galea et al., 2005), is a significant predictor of elevated emotional stress intensity and perception of risk (Grimm et al., 2012), as well as high levels of post-traumatic stress disorder [PTSD, American Psychiatric Association (APA), 2013]. Research findings suggest that exposure to man-made trauma, particularly terror events, leads to more psychological distress then exposure to natural disasters (Norris et al., 2002; Grimm et al., 2012). The effect of exposure to acts of terror on mental health has been investigated by others (for example, Steel et al., 2009, 2014; Durodié and Wainwright, 2019) whose review studies reported higher rates of PTSD and depressive symptoms after such exposure. This association between man-made traumatic events and mental health problems has been reported in studies conducted around the world (Durodié and Wainwright, 2019), and specifically in Israel (Braun-Lewensohn, 2012). Still, victims of terror vary in risk factors for the development of adjustment disorders or PTSD (Mahat-Shamir et al., 2017). Previous experience of stressful events was found to be a significant predictor for developing an adjustment disorder (Mahat-Shamir et al., 2017), increasing vulnerability to developing other adverse psychological effects following terror exposure (Bleich et al., 2006). However, a growing volume of research conducted mainly among adolescents suggests that trajectories of the response to trauma are complicated: a stress response may develop not only following a direct physical exposure or a relationship with a trauma victim (objective exposure) but also after a mere sensation of intense fear (Solomon et al., 2005; Braun-Lewensohn et al., 2009). Moreover, the response to trauma has been associated with personal characteristics; previous studies have linked it to factors such as the individual’s level of neuroticism (Olge et al., 2017), sense of coherence (Braun-Lewensohn et al., 2011; Braun-Lewensohn and Mosseri Rubin, 2013; Braun-Lewensohn and Sagy, 2014), and coping style (Braun-Lewensohn, 2012). Thus, personal characteristics (Venta et al., 2017) may affect resilience, conceptualized by Bonanno (2004, p. 20–21) as the ability to maintain a relatively stable, healthy level of psychological and physical functioning in the face of highly disruptive events, possibly mitigating PTSD development (Zukerman et al., 2017).

One factor that may impact the response to traumatic exposure is place attachment. “Attachment” in the context of the theory of attachment, is described by Bowlby (1988) as an emotional bond with another person that creates a secure base to explore the world. Bowlby claimed that the human infant is innately directed toward seeking proximity and security with significant others (Bowlby, 1982). Such relationships reduce anxiety and enable exploratory behavior (Bowlby, 1982). Attachment relationships were also explored in adulthood, suggesting that intimate relationships might also form attachment bonds (for a review, see Feeney, 2008). Moreover, it was suggested that other relationships may meet the definition of attachment; several authors have suggested that belief in a perceived relationship with G-d also constitutes an attachment relationship (Kirkpatrick, 1994; Granqvist et al., 2010). Attachment relationship characteristics that include proximity-maintaining behavior and security-seeking motives were also hypothesized to explain the function of place attachment (Scannell and Gifford, 2010). More broadly, “place attachment” refers to the long-term bonding that occurs between individuals and meaningful places in their lives (Scannell and Gifford, 2010). The concept refers to the intersection between one’s personal characteristics (both individual and group level), psychological processes (emotional, cognitive, and behavioral) and the nature of the place itself (e.g., geographic range, physical or social spaces) (Billig et al., 2016). Billig (2006) found that people who live in contested Israeli residential areas (sometimes referred to as “settlements”) which have high exposure to terror events, have a strong tendency to stay in the area, exhibit firm ideologies of “holding onto the land,” profess profound religious faith, report a strong attachment to the area and their home, and possess a perception of low security risk. Moreover, living in such areas was associated with higher levels of obligation, cooperation, group discipline, social involvement, and mutual aid than living in a city (Sofer and Applebaum, 2006; Friedman and Billig, 2018). Sense of place, or place attachment, was found to be higher for religious residents in small, compared to large, “settlements” (Casakin and Billig, 2009; Casakin et al., 2015).

Interestingly, terror exposure, such as the kind seen in Israeli “settlements,” may elicit both negative and positive changes in individuals and communities. Terror Management Theory (TMT) posits that death anxiety contributes to ingroup solidarity and hostility toward people living outside the group (Barberia et al., 2018). Moreover, it has also been postulated that cultural and religious practices reduce death anxiety by effecting a sense of self; the individual’s sense of self is anchored in the notion of being a part of the culture itself, above and beyond the existence of their physical selves. There is also a tendency to increase one’s sense of self-esteem through participating in culturally influenced activities, such as religious practices (e.g., communal prayer, religious ceremonies). The use of these practices, conceptualized as “defense mechanisms,” is associated with increased residential attachment and sense of ideology (Friedman and Billig, 2018).

Several research findings suggest that being religious may have a protective effect for individuals exposed to terrorism (Levav et al., 2008; Casakin and Billig, 2009; Korn and Zukerman, 2011; Zukerman and Korn, 2014; Zukerman et al., 2016, 2017). For example, students who define themselves as secular reported higher rates of avoidance behavior following terror event exposure (Zukerman et al., 2016). The reasons behind this variability in response to terror are not yet well understood. However, it is possible that religiosity may moderate the effects of trauma through cognitive schemas; religious cognitive schemas have been found to affect one’s “world assumption”- the basic perception of the world and self (Janoff-Bulman, 1989)-and may do so by creating a “protective shield” that prevents negative effects resulting from undergoing an extremely negative experience (Zukerman and Korn, 2014). Indeed, religious coping, conceptualized as drawing upon religious beliefs and practices in order to understand and deal with life stressors (Pargament, 1997), has been suggested as a key factor influencing adaptation after trauma exposure (Laufer and Solomon, 2011; Zukerman et al., 2016, 2017); it can manifest as either positive or negative. Positive religious coping, including benevolent religious appraisal, praying, and seeking spiritual support and connection with G-d, has been associated with better adjustment to stressful events and reductions in post-traumatic stress symptomatology (Pargament et al., 2000; Harris et al., 2008; Chan and Rhodes, 2013). Such positive coping may elevate feelings of support from “the divine” or from members of one’s religious community (Bryant-Davis and Wong, 2013). Previous research also associated higher levels of religiosity with less negative religious coping (religious discontent, questioning religious beliefs, faith, and practices) after trauma, thus reducing religious conflict (also called spiritual struggle), and probably elevating certainty regarding one’s perceptions of the world (Zukerman et al., 2016, 2017). While not an exclusively “religious” practice an additional postulation about protective effects from trauma is related to mindfulness, conceptualized as the ability to purposely bring one’s attention to the present moment in a non-judgmental manner (Kabat- Zinn, 2013). This technique, promoting the idea that thoughts are not facts, is commonly used for reducing post-traumatic stress symptoms (Boyd et al., 2018). Mindfulness may have religious associations, particularly to Buddhist meditative practices (Kopel and Habermas, 2019) but also to other religions such as Christianity and Judaism (Kopel and Habermas, 2019; Niculescu, 2020). Thus, religiosity may also affect coping after trauma by promoting practicing spiritual mindfulness, as was suggested by some studies (Rudaz et al., 2020).

Religion has been also conceptualized to affect one’s “sense of place,” the thoughts and feelings we have about a place we consider special or unique for us and to which we feel we belong and are attached (Casey, 2001). It is also considered a subjective environmental experience consisting of cognitive, emotional, and behavioral aspects (Jorgensen and Stedman, 2001). The level of “sense of place” (measured by participant responses to a series of statements such as “I think the physical space of where I live is aesthetic”; “I feel secure living in this place”; “I am involved in social and cultural activities where I live”) was reported to be significantly higher among religious, than secular, residents (Casakin and Billig, 2009; Casakin et al., 2015). However, sense of place, alternatively called “place attachment,” has been studied mainly among religious groups with robust ideologies such as Jewish “settlers” in the west bank of Judea and Samaria; such research indicated usually higher levels of place attachment among the more religious individuals in these groups (Billig, 2006; Levav et al., 2008).

According to Ahmed (2016), religious identities assist in establishing trust, openness, and commonality. Religiosity has also been shown to affect other measures of well-being such as depression. Generally, higher levels of religiosity have been associated with better well-being (Koenig et al., 2001) and lower levels of depression (for a review, see Smith et al., 2003; Braam and Koenig, 2019; Davenport and McClintock, 2021). However, the literature indicates a complex relationship pattern between one’s level of religiosity and use of religious coping on mental health indices; both positive and negative religious coping can lead to either improved or reduced well-being, respectively (Ano and Vasconcelles, 2005). Specifically, religious conflict or spiritual struggle (conceptualized as tension and strain about spiritual issues within oneself, with other people, and with the divine; Pargament et al., 2005) may lead to reduced well-being (Rosmarin et al., 2009) and was associated with greater depression (Exline et al., 2000). For example, a recent study examining the relationship between religious involvement and depression among a sample of graduate students indicated that spiritual instability and disappointment in God were distinct predictors of depression (Paine and Sandage, 2017) and demonstrated that religion can be positively or negatively associated with mental health depending on the nature of involvement. Thus, it appears important to examine the possible influence of religiosity on emotional and behavioral adjustment after terror event exposure.

Previous research findings suggest that resilience to traumatic event exposure is affected by both personal and communal characteristics. Previous research evidence has stressed the concept of community resilience as a key factor influencing the reaction to disasters and man-made traumatic events (Eshel and Kimhi, 2016; for a review, see Mayer, 2019). In studies conducted in Israel, community size and affiliation (smaller vs. larger, rural communal vs. urban, Braun-Lewensohn and Sagy, 2014), and level of religiosity (Levav et al., 2008) were associated with higher resilience to the psychological effects of terror attacks. Of particular interest, individuals from certain communities have been found to be more resilient than others to developing post-traumatic stress symptoms after traumatic experiences (Korn and Zukerman, 2011; Zukerman and Korn, 2014; Zukerman et al., 2016, 2017).

The first purpose of this study is to examine possible differences in resilience [as expressed in levels of depressive symptoms, avoidance behavior, and psychosomatic (PS) symptoms] among groups from three different residential areas and community types: (1) Ariel- a city located in Judea and Samaria outside the Green Line (the pre–1967 Israeli border), whose population is mostly secular and temporary student residents (Israel Central Bureau of Statistics, 2018). (2) Small communities in Judea and Samaria (outside the Green Line), and (3) Cities and communities within the Green Line (what will be called “other places in Israel”). The second purpose is to identify factors that account for any identified variance in outcome variables, particularly depressive symptoms. Since our aim was to compare groups with different levels of residence attachment, and in order to accentuate group differences among those residing in Ariel, only those temporarily residing in the city (likely due the need to be close to the university) were included. We hypothesized that: (1) The three groups would differ in characteristics such as religiosity and residence attachment, as well as their level of reported terror exposure. Among students living in Ariel, resilience was expected to be lower due to their temporary residence and lesser religiosity compared to students living permanently in small settlements in Judea and Samaria (Billig, 2006; Zukerman and Korn, 2014; Zukerman et al., 2016, 2017); and (2) Greater residence attachment and level of religiosity would be associated with greater resilience (less depressive symptoms, less avoidance behavior, and less PS symptoms), despite a higher level of terror exposure (Casakin and Billig, 2009; Casakin et al., 2015).

## Materials and Methods

### Participants

A total of 1,442 undergraduate students participated in the study, representing about 20% of all enrolled undergraduate students at one university; the data from twenty-three participants were subsequently removed from the analysis due to significant multiple or missing values. Therefore, the final sample consisted of 1,413 undergraduate students from Ariel University (62.7% female; *M*_age_ = 26.5; SD = 6.03). The study was conducted within all four university departments after obtaining departmental approval: Health Sciences, Natural Sciences, Social Sciences and Humanities, and Engineering.

### Procedure

Approval by the university ethics committee was obtained prior to study onset. All students participating in the study gave their active informed consent by signing a short declaration before completing a questionnaire. Research staff entered all classrooms of four university departments- Health Sciences, Natural Sciences, Social Sciences and Humanities, and Engineering − and distributed the questionnaire to all students present during the last 10 min of class. No participant sampling was utilized for this study. Estimated response rate was almost 90% of students presented in the classrooms. After the class was scheduled a head of time with the lecturer, the research team entered during the 5 last min of the lesson, presented the topic of the study and read aloud all the first page of instructions before handing out the printed questionnaires. The first page contained the information telling them they were allowed to refuse to participate in the study with no explanation needed and they were allowed not to answer all the questions if they didn’t want to. The lecturer was not present in the classroom during distribution of the questionnaires.

### Research Tool

The research tool utilized was a structured, self-report, anonymous questionnaire whose questions were excerpted from three validated sources: (1) Jessor’s Survey of Personal and Social Development (Jessor et al., 2003). Jessor’s questionnaire was translated from English to Hebrew, then back-translated and validated, during a pilot project implemented in 2009. Stress items originated from Jessor’s questionnaire. (2) The Israeli Health Behavior in School-aged Children (HBSC) study headed by the World Health Organization (WHO; Harel-Fisch et al., 2006). Items related to subjective sense of insecurity, avoidance behavior after terror events, and psychosomatic symptoms originated in Hebrew from the Israeli HBSC questionnaire. (3) Beck Depression Inventory (BDI-II; Beck et al., 1996) was used to excerpt the depressive symptom items. Detailed description of the survey’s origin has been previously published (Korn et al., 2013) including information about the questionnaire’s development and methodology.

### Socio-Demographic Characteristics

Information regarding gender, age, family status, and socioeconomic characteristics (mother/father education and monthly family income) was obtained from the self-report questionnaire.

### Independent Variables

The information obtained regarding sociodemographic characteristics was:

#### Religious Orientation

Choices were: (1) Secular, (2) Traditional (perceive themselves as neither strictly religious nor secular, observe selective commandments and customs considered clear symbols of tradition yet do not necessarily commit to wholesale observance of traditional Jewish law), (3) Religious, (4) Ultra-Orthodox (UO), or (5) Other (Israel Democracy Institute, 2019). For further analysis, values 3 and 4 were combined, and the choice of value 5 (Other; 1.4%) was recoded as missing.

#### Place of Residence

Two questions were used to create groups according to “place of residence.” The first question was, “Where is your permanent place of residence?” Values were: (1) Ariel, (2) Another place in Judea and Samaria, (3) A different place in Israel, or (4) Not in Israel, in another country (6 participants, recoded as missing). For those who indicated living in Ariel, we checked by cross-frequent tabulation other questions related to the type of housing in which participants were living. Values were: (1) Student dormitory or village, (2) Home with your parents. (3) House/apartment you own, (4) Rental house/apartment, and (5) Elsewhere. Those living with their parents or in a house/apartment they owned in Ariel (44 participants) were removed from the analysis to isolate only those temporarily residing in Ariel. In this way, the three groups for “place of residence” were: (1) Ariel- representing temporary students living in dormitories in Ariel. (2) Judea and Samaria- representing students living in the area permanently, and (3) Different places in Israel- representing most of the undergraduate students living permanently in many Israeli cities and settlements inside the Green Line.

#### Residence Attachment

Participants were asked, “To what extent do you feel emotionally attached to your current place of residence?” Values were: (1) Completely attached, (2) Attached, (3) Partially attached, (4) Not attached, and (5) Completely not attached. For further analysis, the scale was reversed and values 1–3 were combined into “Not attached” and values 4 and 5 were combined into “Attached.”

#### Objective Exposure to Terror Events Scale

This scale, used previously (Hamama-Raz et al., 2008), contains five items that assess direct or vicarious exposure to terror events (“A terror event happened where I live”/“Someone not close to me that I know was involved in a terror attack”/“Someone close to me was involved in a terror attack”/”Someone close to me was hurt in a terror attack”/“I was close to getting hurt or got hurt in a terror attack”). Values were: 0. Never happened, 1. Happened, but I was not scared, 2. Happened, and I was a little scared, 3. Happened, and I was scared, or 4. Happened, and I was very scared. Terror event exposure was formed by summation of five new items created by dichotomizing responses to either “0 (never happened)” or “1 (happened, regardless of the response’s affective intensity level).” These items were then summed into one grouped item that ranged from 0 = “less exposed” to 5 = “more exposed”; Cronbach’s alpha = 0.78.

#### Avoidance Behavior After Terror Events Scale

This scale, used previously (Korn and Zukerman, 2011), contained six items related to behavioral changes following a terror event representing avoidance behavior (“I have modified my social life as a result of the terror attacks”/“I avoid traveling to certain places as a result of the terror attacks”/“I hang out less as a result of the terror attacks”/“I feel I concentrate less as a result of the terror attacks”/“I didn’t arrive to school as a result of the terror attacks”/“I need to use other roads rather than those I used before because of the security situation”). Values were: 0. Never happened, 1. Happened, but I was not scared, 2. Happened, and I was a little scared, 3. Happened, and I was scared, or 4. Happened, and I was very scared. Frequency of avoidance behavior was formed by summing six new items created by dichotomizing responses to either “0 (never happened)” or “1 (happened, regardless of the response’s affective intensity level).” These six items were summed to one scale that created a spectrum of avoidance associated behavior (higher grades indicate more avoidance behavior); Cronbach’s alpha = 0.71.

#### Psychosomatic Symptoms

Participants were asked eight questions regarding how often they have had the following in the last 6 months (Currie et al., 2008): headache, stomachache, backache, feeling low, irritability or bad temper, nervousness, difficulties getting to sleep, and dizziness. Responses ranged on a Likert scale from 1 = “rarely or never” to 5 = “about every day.” Summed items ranged from 8 = “rarely/never” to 40 = “about every day”; Cronbach’s alpha = 0.80.

#### Residential Stress

Participants were asked, “Over the past month, how much stress did you feel because of your place of residence?” Values were: (1) Not at all, (2) A little bit, (3) Much, and (4) Very much (Jessor et al., 2003).

### Dependent Variable

#### Depressive Symptoms

Four items were taken from the Beck Depression Inventory (BDI – II, Beck et al., 1996) to measure depressive symptoms. The BDI is a 21 item self-report questionnaire that is widely used and has high reliability (internal consistency 0.89–0.94) and validity (as expressed by high correlation with other depression related self-rating scales) (Beck et al., 1996). The four items used in this study included questions regarding sadness, failure, discouragement, and dissatisfaction. Values ranged from 1 = “I do not have this feeling” to 4 = “I have a strong feeling of this.” Items were summed and ranged from 4 = “less depressed” to 16 = “more depressed”; Cronbach’s alpha = 0.73.

### Analysis

IBM SPSS-21 was used for initial descriptive statistics. Cross tabulation frequencies were used to present percentages in each residential group with Pearson chi-square testing for differences between groups (Table 1), while one-way ANOVAs were used with *post hoc* testing to present the mean differences between the four residential groups (Tables 1, 2). In order to evaluate the possible association between terror event exposure and the outcome variable (depressive symptoms), a Multinomial Logistic Regression with Adjusted Odds Ratios (AOR) values was conducted. In this analysis, AORs are odds ratios that control for other predictor variables in a model (gender, age, family status, mother education, father education, and religion) and give an idea of the dynamics between several independent variables in the multiple regressions. Odds ratios measure the association between exposure to an outcome and represents the probabilities, or odds, that an outcome will occur given a particular exposure, compared to the odds of the outcome occurring in the absence of that exposure in one group (exposure group) compared to a reference group. An odds ratio of “1” represents no association between exposure to outcome. Odds ratios higher than 1 represent higher probabilities of an outcome in the exposure group relative to the referent group (thus an odds ratio of 1.5 indicates the probability of an outcome in the exposure group is 150% compared to the reference group). Odds ratios lower than 1 suggest the probability of an outcome in exposure group are lower than among the reference group (thus an odds ratio of 0.75 indicates the probability of an outcome in the exposure group is 75% compared to the reference group). Table 3 shows outcomes from Multinomial Logistic Regression with AOR values. In this analysis, “cities inside the Green Line” and “communities inside the Green Line” were combined into one group named “other places in Israel.” Two statistical models were run in one regression analysis: Model 1 with residence in “small communities in Judea and Samaria” as a referent group (control group) to “Ariel” and “other places in Israel” as study groups; meaning, if the odds of an exposure to each variable are higher than 1, the probability of having depression symptoms are higher in Ariel or “other places in Israel” than in “Judea and Samaria.” Model 2 presented outcomes in the study groups of “Judea and Samaria” and “other places in Israel” with “Ariel” as a referent group.

**TABLE 1:** Socio-demographic variable differences by residential groups (weighted for gender).

		Ariel- city in Judea and Samaria		Community settlements in Judea and Samaria		Other places in Israel		*p*	All sample	
						
		*n*	*M(*SD*)*	*n*	*M(*SD*)*	*n*	*M(*SD*)*		*N*	*M(*SD*)*
Age		278	24.9 (3.1)	234	27.0 (6.4)	830	9.62 (6.0)	0.002	1341	26.5 (5.6)
Mother education		269	6.0 (1.7)	225	6.3 (1.7)	778	55 (1.9)	0.000	1271	5.7 (1.8)
Father education		258	5.7 (2.0)	221	5.9 (1.8)	746	35 (2.0)	0.000	1225	5.5 (2.0)
All sample (%)		326	23.1	237	16.8	508	0.26		1413	100.0
		*n*	%	*n*	%	*n*	%		*N*	%
Gender* (%)	Females	149	45.7	107	45.1	504	52.9	0.020	706	50.0
	Males	177	54.3	130	54.9	400	7.14		707	50.0
Family status	Single	245	88.8	94	40.5	611	2.47		509	71.4
	Married	30	10.9	134	57.8	921	3.32	0.000	356	726.0
	Divorced	1	0.4	4	1.7	19	32.0		24	1.8
	Widow/er	0	0	0	0	1	0.1		1	0.1
Family monthly income (%)	Above average	102	37.2	85	37.9	318	0.893		505	938.0
	Average	123	44.9	92	41.1	349	6.34	0.585	564	43.5
	Below average	49	17.9	47	21	133	6.61		229	617.0
Religion (%)	Secular	113	40.9	30	13.1	359	244.0	0.000	250	138.0
	Traditional	65	6.32	34	14.8	211	0.62		031	523.0
	Religious/UO	98	35.5	561	72.1	243	9.92		650	0.483

*UO, ultra-orthodox.*

**TABLE 2:** Study variables’ mean differences by residential groups (weighted for gender).

Measures		Ariel- city in		Community settlements		Other places		*p*	All sample	
		Judea & Samaria		Judea & Samaria		in Israel				
					
	values	*n*	*M(*SD*)*	*n*	*M(*SD*)*	*n*	*M(*SD*)*		*n*	*M(*SD*)*
Depressive symptoms	4-less depressed…	264	4.9 (1.6)	223	4.5 (1.1)	768	4.8 (1.4)	0.009	1256	4.8 (1.4)
	16-more depressed									
Exposure to terror	0-less exposed…	243	1.7 (1.6)	211	2.4 (1.6)	699	1.1 (1.4)	0.000	1153	1.5 (1.6)
	5-more exposed									
Residence attachment	1-not at all attached…	279	3.0 (1.0)	233	3.7 (0.9)	825	3.7 (1.0)	0.000	1337	3.6 (1.0)
	5-very attached									
Avoidance behavior	6-less avoidance…	238	9.2 (3.3)	211	8.7 (2.8)	688	9.6 (3.7)	0.000	1138	9.4 (3.5)
	30-more avoidance									
Psychosomatic symptoms	8- never…	261	17.3 (5.7)	228	16.0 (5.1)	788	17.4 (5.9)	0.011	1277	17.1 (5.8)
	40-almost everyday									
Residential stress	1-not at all…	278	1.7 (0.8)	233	1.5 (0.7)	821	1.7 (0.8)	0.015	1333	1.6 (0.8)
	4-very much									

ANOVA significance *p < 0.05, **p < 0.01, and ***p < 0.001.

**TABLE 3:** Adjusted odds ratios (AOR) for associations between depression symptoms and study variables by place of residence (weighted for gender; multi-nominal logistic regression).

		Model 1						

		Depression symptoms						
Scales median split		AOR J and S	AOR Ariel	CI (Low-High)		AOR other	CI (Low-High)	
		(Ref)				Place		
Gender	1 = males, 2 = females	1.00	1.06	0.64	1.75	0.70	0.46	1.07
Age	18–25 = 0; 26–66 = 1	1.00	1.22	0.72	2.05	0.73	0.46	1.14
Family status	0 = married/coupled; 1 = not married/coupled	1.00	0.14***	0.08	0.26	0.28***	0.18	0.43
Mother education	0 = higher; 1 = lower	1.00	0.85	0.48	1.47	0.71	0.44	1.13
Father education	0 = higher; 1 = lower	1.00	1.38	0.79	2.39	0.93	0.58	1.48
Religion	0 = religious; 1 = traditional, secular	1.00	0.38***	0.23	0.62	0.31***	0.20	0.48
Exposure to terror	0 = low exposure; 1 = high exposure	1.00	2.12***	1.32	3.40	3.21***	2.15	4.78
Residence attachment	0 = attached; 1 = not attached	1.00	0.31***	0.19	0.49	0.94	0.63	1.40
Avoidance behavior	0 = less; 1 = more	1.00	0.89	0.55	1.44	0.84	0.55	1.26
PS symptoms	0 = less; 1 = more	1.00	0.96	0.58	1.59	1.00	0.65	1.54
Residential stress	0 = less; 1 = more	1.00	0.77	0.48	1.24	0.82	0.55	1.23

		**Model 2**						

		**Depression symptoms**						
**Scales median split**		**AOR Ariel (Ref)**	**AOR J and S**	**CI (Low-High)**		**AOR other place**	**CI (Low-High)**	

Gender	1 = males, 2 = females	1.00	0.94	0.57	1.54	0.66*	0.45	0.96
Age	18–25 = 0; 26–66 = 1	1.00	0.81	0.48	1.38	0.59**	0.40	0.88
Family status	0 = married/coupled; 1 = not married/coupled	1.00	6.79***	3.83	12.03	1.91*	1.14	3.19
Mother education	0 = higher; 1 = lower	1.00	1.17	0.67	2.04	0.83	0.55	1.26
Father education	0 = higher; 1 = lower	1.00	0.72	0.41	1.25	0.67	0.44	1.01
Religion	0 = religious; 1 = traditional, secular	1.00	2.62***	1.59	4.34	0.82	0.56	1.20
Exposure to terror	0 = low exposure; 1 = high exposure	1.00	0.47***	0.29	0.75	1.51**	1.05	2.17
Residence attachment	0 = attached; 1 = not attached	1.00	3.20***	2.01	5.09	3.01***	2.12	4.28
Avoidance behavior	0 = less; 1 = more	1.00	1.122	0.69	1.81	0.94	0.65	1.35
PS symptoms	0 = less; 1 = more	1.00	1.03	0.62	1.70	1.03	0.71	1.51
Residential stress	0 = less; 1 = more	1.00	1.28	0.80	2.05	1.06	0.74	1.52
Nagelkerke R^2^		30.7%						
*N*		928						

ANOVA significance *p < 0.05, **p < 0.01, and ***p < 0.001.

*PS, Psychosomatic.*

## Results

Table 1 shows socio-demographic characteristics by residential group, in the sample weighted for gender. No significant group differences were observed in monthly income, but significant differences were shown regarding age (*p* < 0.01), parental education (*p* < 0.001), gender (*p* < 0.05), family status (*p* < 0.001), and religion (*p* < 0.001). Participants living in Ariel were younger compared to the entire sample (*M* = 24.9, SD = 3.1 vs. *M* = 26.5, SD = 5.6, *p* < 0.01). Participants’ parental mean years of education were the lowest among students from “other places in Israel” compared to the entire sample (Mother- *M* = 5.5, SD = 1.9 vs. *M* = 5.7, SD = 1.8, *p* < 0.001; Father- *M* = 5.3, SD = 2.0 vs. *M* = 5.5, SD = 2.0, *p* < 0.001). Significant differences (*p* < 0.001) were also found regarding family status and religion between residential groups, with a higher frequency of single participants living in Ariel (88.8% compared to 45.3% in “community settlements at Judea and Samaria,” and 74.2% in “other places in Israel”), and a higher frequency of “religious” participants living in community settlements in Judea and Samaria (72.1% compared to 35.5% in Ariel and 29.9% in “other places in Israel”).

Table 2 presents the mean differences in study variables between residential groups, weighted for gender. For all variables, participants who lived in “small community settlements in Judea and Samaria” exhibited greater well-being (less depressive symptoms, avoidance behavior, and PS symptoms) compared to the groups from other residential areas: they reported less avoidance behavior (*M* = 8.7; *p* < 0.001), less psychosomatic symptoms (*M* = 16.0; *p* < 0.05), less residential stress (*M* = 1.5; *p* < 0.05), and less depressive symptoms (*M* = 4.5; *p* < 0.001) compared to all others (9.4, 17.1, 1.6, 4.8, respectively). This group also exhibited higher residential attachment (*M* = 3.7 compared to 3.0 in Ariel, or 3.6 in the total sample) despite higher objective exposure to terror events (*M* = 2.4 compared to 1.7 in Ariel, or 1.1 in “other places in Israel”; *p* < 0.000).

Table 3 presents outcomes from a multi-nominal logistic regression of AOR for associations between depressive symptoms and different measures of health and emotional adjustment by place of residence (weighted for gender) in two models. Model 1 presents the results of residents of Ariel and “other places in Israel” with “Judea and Samaria” as a referent group and Model 2 presents the results of residents of “small communities in Judea and Samaria” and “other places in Israel” with Ariel as a referent group. Table 3 clearly shows the four variables strongly and significantly associated with depressive symptoms within the two statistical models – family status, religiosity, exposure to terror, and residence attachment – all significantly contributed to the total explained variance of 30.7%. In Model 1, the probability of participants living in Ariel (compared to those living in small community settlements in Judea and Samaria) to report depressive symptoms was lower if they were: married/coupled (AOR = 0.14; 95% CI = 0.08, 0.26; *p* < 0.001), more religious (AOR = 0.38; 95% CI = 0.23, 0.62; *p* < 0.001), and felt more attached to their place of residence (AOR = 0.31; 95% CI = 0.19, 0.49; *p* < 0.001), while the probability to report depressive symptoms was higher if they were highly exposed to terror (AOR = 2.12; 95% CI = 1.32, 3.40; *p* < 0.001). Similarly, in Model 2, the probability of participants living in small community settlements in Judea and Samaria (compared to those living in Ariel) to report depressive symptoms was higher if they were: not married/coupled (AOR = 6.79; 95% CI = 3.83, 12.03; *p* < 0.001), traditional or secular rather than religious (AOR = 2.62; 95% CI = 1.59, 4.34; *p* < 0.001), less exposed to terror (AOR = 0.47; 95% CI = 0.29, 0.75; *p* < 0.001) and felt less attached to their place of residence (AOR = 3.20; 95% CI = 2.01, 5.09; *p* < 0.001). Therefore, for participants living in small community settlements in Judea and Samaria, compared to those living in Ariel, being single, secular, and less attached to one’s place of residence all raised the probability of reporting higher depressive symptoms. In this same residential group, being less exposed to terror was also associated with higher depressive symptoms, compared to those living in Ariel.

## Discussion

This study focused on understanding depression symptoms reported by undergraduate university students who live in three Israeli residential area types. The first was Ariel, the city in which the university is located; it lies outside the “Green Line” and living there is characterized by a high level of terror exposure. Student residents of Ariel were: young, single, living temporarily in dormitories or rented apartments, more secular, and have less residential attachment; these findings are corroborated by previous study results (Korn and Billig, 2013). The second residential area type was small community settlements in Judea and Samaria (also outside the Green Line). Participants in this group were older, mostly married, more religious, and feel more attached to their place of residence compared to the other groups. The third residential area type consisted of “other places in Israel” – both rural and urban environments inside the Green Line, in which most of the university students lived, and therefore served as reference group. Associations were examined between several risk and protective factors among students, such as residential attachment, level of terror event exposure, cultural and socio-demographic variables, and the level of symptoms of depression. We hypothesized that, among students living in Ariel, resilience would be lower due to their temporary residence and lesser religiosity compared to students living in small settlements in Judea and Samaria. We also predicted that, among our entire sample, residence attachment would be positively associated with resilience- meaning less depressive symptoms, avoidance behavior, and PS symptoms, despite higher level of exposure to terror events.

The effect of exposure to terror events on mental health has been studied extensively, indicating an association between exposure to terror, rates of PTSD, and levels of depressive symptoms (Steel et al., 2009, 2014; Grimm et al., 2012; Slone and Mann, 2016; Mahat-Shamir et al., 2017; Durodié and Wainwright, 2019). In addition, a previous study among Israeli students showed significant differences between these three residential area groups both in socio-demographic measures and in health and risk behaviors (Korn and Billig, 2013). In continuation of their work, in the current study, the comparison of students living in community settlements in Judea and Samaria to those living in Ariel or other places in Israel suggests that students living in Judea and Samaria settlements report better well-being. Our findings indicate they report fewer symptoms of depression, lower rates of reported psychosomatic symptoms, less stress resulting from their places of residence, and less avoidance behavior, in spite of reporting greater exposure to terror incidents. Our findings suggest that some factors may enhance resilience to relatively high rates of traumatic event exposure, including: being married or in a relationship, greater religiosity, and higher residence attachment.

The protective effect of religiosity on the impact of exposure to terrorism is known and was previously studied (Levav et al., 2008; Casakin and Billig, 2009; Korn and Zukerman, 2011; Zukerman and Korn, 2014; Zukerman et al., 2016, 2017). In our study, depressive symptoms were lower among religious participants living in Ariel compared with those living in community settlements in Judea and Samaria (AOR = 0.38; 95% CI = 0.23, 0.62; *p* < 0.001), and higher among traditional or secular participants (AOR = 2.62; 95% CI = 1.59, 4.34; *p* < 0.001) living in small community settlements in Judea and Samaria compared to those living in Ariel. These findings suggest a new, interesting hypothesis regarding the mechanism by which religiosity may enhance resilience after trauma. Since religion was associated with forming closed-knit communities (Frounfellker et al., 2020) and higher levels of community involvement (Theodori and Chothiakadavil, 2019), their higher level of community involvement and place attachment, and not their religiosity *per se*, may lead to enhanced resilience. This notion is partly corroborated by research findings attributing positive effects of religion on mental health among those participating in religious communities (VanderWeele, 2017). However, this was not tested in the current study and should be explored in future research. Additionally, our findings suggest that religious people living in a mostly secular place tended to report less depression, while secular individuals living in a mostly religious place tended to report greater depressive symptoms. One possible explanation for this finding is that being different from other community members reduces the level of attachment to one’s community and possibly leads to greater stress and depression symptoms. This issue should be explored in future studies.

Previous studies have shown place attachment to be significantly higher among religious than secular residents (Billig, 2006; Billig et al., 2006; Casakin et al., 2015). Compared to students living in other residential areas in Judea and Samaria and within the 1949 armistice borders of Israel, students living mostly temporarily in Ariel reported less residential attachment. Given that all university students in our final sample were living in dormitories or rented apartments near the university primarily during the period of their studies, place attachment may be less robust when one’s residence is perceived as temporary. The similarity in level of place attachment between the students living permanently in Judea and Samaria and those living in other places in Israel may be explained by a combination of components that affect emotional attachment to one’s residence. Having a permanent place of residence may increase one’s place attachment by facilitating a perception of greater quality of life (Mao et al., 2015) and closeness to family and friends (Wiles et al., 2009), as well as due to ideological reasons (Billig, 2006) such a religious connection to one’s land and/or patriotism. Place attachment can bridge geographic and social boundaries (Gurney et al., 2017). One possible important implication of our findings is that local or academic community intervention programs aimed to promote place attachment may have particularly beneficial effects on undergraduate students in Ariel, who may be more highly exposed to terror, relative to students living in other places in Israel, despite being less exposed to terror activity than other locations in Judea and Samaria. However, further research is needed to strengthen these findings. If universities’ and institutions’ student welfare services are unaware or unable to dedicate resources to this concern, it may be that it can relatively easily be addressed through personal strengthening of social cohesion and mutual responsibility.

In this study, demographic and cultural characteristics–family status and religiosity–as well as residence type, place attachment, and high frequency of exposure to terror incidents, significantly explained 30% of the variance in depressive symptoms among students. Our findings are corroborated by previous research findings associating marital status and religious affiliation with variance in reported levels of depressive symptoms (Levav et al., 2008; Korn and Zukerman, 2011; Zukerman and Korn, 2014). These findings, suggesting that greater religiosity, higher place attachment, and being married or in a relationship, are protective factors within environments that are highly exposed to terror, finds support from Bowlby’s attachment theory (Bowlby, 1988) and highlights the potential importance of creating better attachment in increasingly isolated societies. Moreover, they support the assumption that, in shadow of terror attacks, more community resilience (Eshel and Kimhi, 2016) leads to better outcomes. Accumulating research evidence suggests that community resilience might lead to diminished negative consequences following disaster (for a review, see Mayer, 2019), particularly, in face of terror attacks (Braun-Lewensohn and Sagy, 2014; Eshel and Kimhi, 2016). These findings further support Durkheim’s important claim (Durkheim, 1897, in: Condorelli, 2016) that in a situation of protracted external threat, community resilience is strengthened.

Along with previous studies, our findings indicate that among participants living in community settlements in Judea and Samaria, being single, secular, and less attached to one’s residential area raises the probability of reporting higher depressive symptoms. However, the findings also suggest that, among this population, being less exposed to terror was associated with higher depressive symptoms. It is possible that, in small community settlements in Judea and Samaria that regularly suffer from terror events, higher exposure promotes higher resilience to depression. This finding may be corroborated by previous studies on this population and similar populations living in “risky” areas (Billig, 2006; Casakin et al., 2015) that indicate “community resilience” positively affects residents’ quality of life, despite the danger to their lives, through social support and cohesion, or through in-group solidarity as suggested by TMT (Barberia et al., 2018). Students living in the Judea and Samaria region may have become accustomed to the relative routine reality of terror events (Tuval-Mashiach et al., 2004) thus mitigating the potential impact, such as emergence of depressive symptoms. It may therefore be reasonable to expect that students who are seldom exposed to terror attacks may report higher levels of depressive symptoms when eventually exposed to such events, compared to those who frequently experience terror and consequently may have developed mental and environmental coping mechanisms.

The study’s strengths and limitations provide insights for future research. The large sample, a particular strength of the study, allowed comparisons of different residential groups and enabled the use of statistical controls for multiple pertinent covariates. In contrast, a potential study confound is the lack of verification that terror events reported by participants actually occurred in their specific place of residence. In addition, the study is limited by the use of self-report questionnaires to assess depressive symptoms, and the use of only selected items from the BDI (Beck et al., 1996), rather than the full instrument. Similarly, due to a desire to restrict its length, only components of instruments were used to compose the research questionnaire. In future research that focuses on these aspects, the use of full scales would be an advantage. Moreover, religious orientation was used as a nominal differentiation and might not be the best indicator of a person’s religiosity, as it doesn’t address experiences, attitudes or practices. Additionally, the study sample is comprised of young adults, while the other age groups were previously explored and should be examined in future studies; indeed, more studies should test the associations between exposure to terror, age, and mental health. Lastly, the findings do not allow understanding of all aspects of place attachment components, and as an observational study, we cannot control all possible influences on the results. For example, people who reside in small settlements in Judea and Samaria may also be ideologically very different from other populations. Hence, it may not be only religiosity and residence attachment, but also the feeling of being “right” in the context of an ideological struggle, that leads to improved resilience.

While this study was based on an Israeli population with specific cultural characteristics, resilience factors such as the degree of belonging to a residential area and the degree of religiosity, as well as risk factors such as exposure to terror incidents, can significantly predict outcomes in other parts of the world as well. The findings of this study are only observational and do not indicate causality between variables. Future research efforts should be devoted to better assessing difficulties faced by students from different backgrounds in order to enable assistance in coping with traumatic environmental stressors (such as the risk of terror activity), in addition to the usual tensions associated with an academic environment. Our findings also stress the importance of developing intervention techniques aimed at prevention of negative outcomes of terror exposure, including depressive symptoms, by facilitating a greater sense of belonging to one’s place of residence.

## Data Availability Statement

The datasets presented in this article are not readily available because of ethical/privacy reasons. Requests to access the datasets should be directed to GZ, gilzu@ariel.ac.il.

## Ethics Statement

The studies involving human participants were reviewed and approved by the Ariel University Ethics Committee. The patients/participants provided their written informed consent to participate in this study.

## Author Contributions

LK conceived and designed the study and performed the statistical analysis. MB and GZ assisted in writing and reviewing the current manuscript. All authors contributed to the article and approved the submitted version.

## Conflict of Interest

The authors declare that the research was conducted in the absence of any commercial or financial relationships that could be construed as a potential conflict of interest.

## Publisher’s Note

All claims expressed in this article are solely those of the authors and do not necessarily represent those of their affiliated organizations, or those of the publisher, the editors and the reviewers. Any product that may be evaluated in this article, or claim that may be made by its manufacturer, is not guaranteed or endorsed by the publisher.
